# Detecting archaic introgression using an unadmixed outgroup

**DOI:** 10.1371/journal.pgen.1007641

**Published:** 2018-09-18

**Authors:** Laurits Skov, Ruoyun Hui, Vladimir Shchur, Asger Hobolth, Aylwyn Scally, Mikkel Heide Schierup, Richard Durbin

**Affiliations:** 1 Bioinformatics Research Centre, Aarhus University, Aarhus C., Denmark; 2 Department of Genetics, University of Cambridge, Cambridge United Kingdom; 3 Wellcome Sanger Institute, Hinxton, Cambridge, United Kingdom; University of Copenhagen, DENMARK

## Abstract

Human populations outside of Africa have experienced at least two bouts of introgression from archaic humans, from Neanderthals and Denisovans. In Papuans there is prior evidence of both these introgressions. Here we present a new approach to detect segments of individual genomes of archaic origin without using an archaic reference genome. The approach is based on a hidden Markov model that identifies genomic regions with a high density of single nucleotide variants (SNVs) not seen in unadmixed populations. We show using simulations that this provides a powerful approach to identifying segments of archaic introgression with a low rate of false detection, given data from a suitable outgroup population is available, without the archaic introgression but containing a majority of the variation that arose since initial separation from the archaic lineage. Furthermore our approach is able to infer admixture proportions and the times both of admixture and of initial divergence between the human and archaic populations. We apply the model to detect archaic introgression in 89 Papuans and show how the identified segments can be assigned to likely Neanderthal or Denisovan origin. We report more Denisovan admixture than previous studies and find a shift in size distribution of fragments of Neanderthal and Denisovan origin that is compatible with a difference in admixture time. Furthermore, we identify small amounts of Denisova ancestry in South East Asians and South Asians.

## Author summary

The genetic history of present-day individuals includes episodes of mating between divergent groups, which have led to 'introgressed' genetic material persisting in modern genome sequences. Perhaps the most notable examples of such events in humans are the introgressions from Neanderthals into non-Africans 50,000 or so years ago, and from a related archaic group known as Denisovans into the ancestors of indigenous people from Papua-New Guinea and Australia. Methods to identify introgressions and the genomic regions that derive from them generally involve the use of reference genome sequences for the source populations. However, there are advantages in having methods independent of reference sequences, both to reduce bias and to detect possible introgression from groups for which we currently lack a reference genome. In this paper we describe such an approach, in a statistical framework which exploits the fact that introgressed regions will contain a high density of genetic variants that are private to the group receiving the divergent material. We apply this method to 89 Papuan genome sequences, estimating times of introgression and initial divergence between archaic and modern humans, and compare it to other related methods.

## Introduction

Archaic introgression into modern humans occurred at least twice (Neanderthals and Denisovans) [1,2] and had a phenotypic effect on humans [3–5]. A substantial amount of Neanderthal and Denisovan genetic material is still present in modern humans and we can learn about archaic populations from studying their genetic variants in humans.

To harness this information a number of methods have been developed to infer segments of archaic ancestry in an individual’s genome. Scanning along the genome, Hidden Markov Models (HMMs)[1,6] and Conditional Random Fields (CRFs)[7] can identify haplotype segments in non-Africans that are both closer to the archaic reference genomes than to Africans, and also longer than expected by incomplete lineage sorting; these are then identified as likely archaic introgressed segments. Another approach is to identify segments with more variants in high linkage disequilibrium (LD) that are unique to non-Africans than expected given a certain demographic scenario [8]. The latest implementations of this method also use an archaic reference genome for refining the set of putative archaic haplotypes [9].

The use of archaic reference genomes for identification of introgressed fragments has drawbacks. First, since the introgressing Neanderthal is closer to the Neanderthal reference genomes (80,000–145,000 years divergence)[10], than the introgressing Denisova is to the Denisova genome (276,000–403,000 years divergence)[1] detecting Denisovan ancestry will be harder. Second, the reliance on having reference genomes implies that the introgression maps need updates whenever more archaic reference genomes are sequenced [10]. Finally, it may be hard to identify potential introgressed segments from an unknown archaic origin, as in the case of the putative archaic introgression into Pygmies [11] and Andamanese islanders [12]. There have been previous approaches that do not use reference genomes, including the initial version of S* [8] and recent methods [13,14].

Here we present a new method for the identification of archaic segments that does not require an archaic reference genome but does require an outgroup population that does not contain admixed genetic material from the archaic population. We implement an HMM that examines the density of variants unseen in the outgroup along individual genomes. Because our method is based on a demographic model incorporating mutation and recombination over time, we can estimate demographic parameters relevant to introgression. We demonstrate with Papuans how we can estimate such parameters and infer more archaic material than previously. Furthermore we can separate the archaic material into Denisovan and Neanderthal components, which display different length distributions in accordance with different admixture times.

### Overview of the model

**Fig 1:**
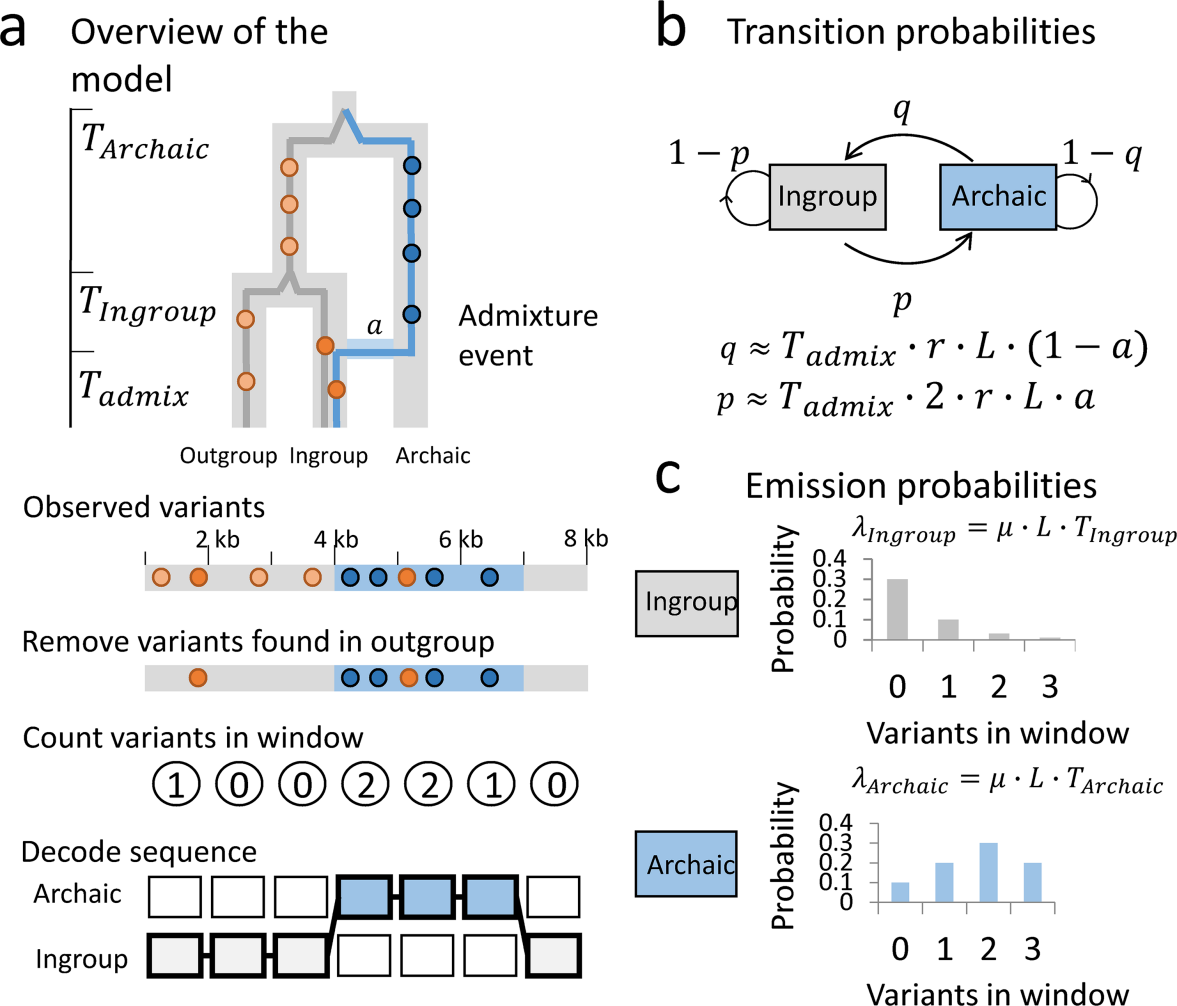
Overview of the model. Illustration on small test dataset. a) An archaic segment introgresses into the ingroup population at time *T*_*admix*_ with admixture proportion *a*. The segments in the ingroup have a mean coalescence time with a segment from the outgroup at time *T*_*Ingroup*_ and an archaic segment has a mean coalescence time with a segment from the outgroup at time *T*_*Archaic*_. Removing all variants found in the outgroup (light orange points) should remove all the variants in the common ancestor of ingroup and outgroup, leaving only private variants that either occurred on the ingroup branch (dark orange) or on the archaic branch (dark blue). This will make the archaic segment have a higher variant density. The genome is then binned into windows of length *L* (here 1000 bp) and the number of private variants is counted in each window. These are the observations and the hidden states are either Ingroup state or Archaic state. When decoding the sequence the most likely path through the sequence is found. b) The transition matrix between the archaic state and ingroup state. c) The emission probabilities are modelled as Poisson distributions with means *λ*_*Ingroup*_ and *λ*_*Archaic*_. It is more likely to see more private variants in the Archaic state than in the Ingroup state.

An archaic genomic segment introgressed into a population is expected to have a high density of variants not found in populations without the introgression. We use a Hidden Markov Model (HMM) to classify genomic segments into states with varying density of such variants. We focus on a scenario where introgression from a deeply divergent archaic population only happened into an ingroup and not the outgroup, see Fig 1A. By removing variants found in the outgroup we can better distinguish introgressed segments from non-introgressed segments based on the density of remaining variants, see Fig 1A. These remaining variants, which we denote private variants (because they are private to the ingroup with respect to the outgroup) can either have occurred on the branch starting from the split of the ingroup and outgroup, or on the introgressing population’s branch. Because the introgressed segments have had a longer time to accumulate variants, they should have a higher density of private variants.

Thus, we define a HMM with two states. The hidden states are Ingroup and Archaic, and the probability for changing state in the Ingroup is *p* and the probability for changing state in the Archaic is *q*, see Fig 1B. The probability of changing state can also be expressed in terms of a constant recombination rate between windows *r* ∙ *L*, the admixture time *T*_*admix*_ and admixture proportion *a*, see Fig 1B.

For practical purposes we bin the genome into windows of length L (typically *L* = 1000 *bp*). The number of private variants observed in a window is Poisson distributed with a rate *λ*_*Ingroup*_ and *λ*_*Archaic*_, respectively where *λ*_*Ingroup*_ = *μ* ∙ *L* ∙ *λ*_*Ingroup*_ and *λ*_*Archaic*_ = *μ* ∙ *L* ∙ *λ*_*Archaic*_, *μ* is the mutation rate, *T*_*Ingroup*_ is the mean coalescence time for the ingroup and the outgroup and *T*_*archaic*_ is the mean coalescence time for the archaic population and the outgroup, see Fig 1C.

We make a correction to the rates to take into account the number of missing bases in a window and the local mutation rate. For window *i* we have $\lambda_{Ingroup}^{i}=\mu_{i}∙L_{i}∙T_{Ingroup}$ and $\lambda_{Archaic}^{i}=\mu_{i}∙L_{i}∙T_{Archaic}$, where *μ*_*i*_ is the local mutation rate and *L*_*i*_ is the number of called bases in a window.

The set of transition parameters *p*, *q* and the Poisson parameters *λ*_*Ingroup*_, *λ*_*Archaic*_ that maximize the likelihood given the observations are found using the Baum-Welch algorithm for an individual genome. These parameters are informative of the mean coalescence times between the ingroup and outgroup and between the archaic and the outgroup, the admixture time and the admixture proportion if we assume a known mutation rate *μ* and a known recombination rate between windows *rL*. Once the set of optimal parameters are found they can be used to decode the genome, using posterior decoding to identify candidate introgressed segments as consecutive regions with posterior probability of coming from the archaic state above some threshold.

Until now we have assumed the data is phased haploid genomes. But to avoid problems with phasing we run this model on unphased diploid genomes. Heterozygous archaic segments will still stand out from homozygous non-introgressed segments. Formally this is equivalent to assuming that homozygous introgressed segments are sufficiently rare that they can be ignored for model fitting. In practice any homozygous archaic segments will have higher private variant density than heterozygous segments, so in the absence of a homozygous HMM state they will be classified with the heterozygous state. We show how to convert model parameters to demographic parameters, both when analyzing haploid and diploid genomes in S1 Dataset.

We note that this method will likely only work in cases where the coalescence time distribution of the ingroup and archaic segments are sufficiently different. This will work better in cases where the variation in the ingroup is a subset of variation in the outgroup so the majority of variation in the common ancestor can be removed, as the case of Non-Africans and Africans.

## Results

### Testing the model with simulations

To investigate the ability of our model to identify archaic (Neanderthal and Denisovan) admixture into Papuans we simulated whole diploid autosomal data using a coalescent simulator, with admixture with an archaic hominin 1,500 generations ago replacing 0–25% of the population–(a script with all demographic parameters is shown in S2 Dataset and a graphical representation of the demography is shown in S1 Fig). We simulated different scenarios to test the effects of running the model on haploid versus diploid data, adding missing data, varying recombination rate and varying a mutation rate.

First, we simulated five individuals where every base in the genome is called equally well and there is a constant recombination rate of 1.2 ∙ 10^−8^ events per basepair per generation. We call this dataset the ideal data. Second, we simulated five individuals with missing data (using the repeatmask track for the human reference genome hg19 [15]) and variations in local recombination rate (using HapMap phase II [16]) to test the effect of missing data and recombination. Third we add variations in local mutation rate to the second scenario as described in the materials and method section. We binned all genomes into bins of 1000 bp, and removed all variants found in any of 500 simulated Africans, 100 simulated Europeans and 100 simulated Asians. We train the model on both haplotype data and unphased diploid genotype data for the simulated individuals. The latter is similar to situations where phased data is not available.

**Fig 2:**
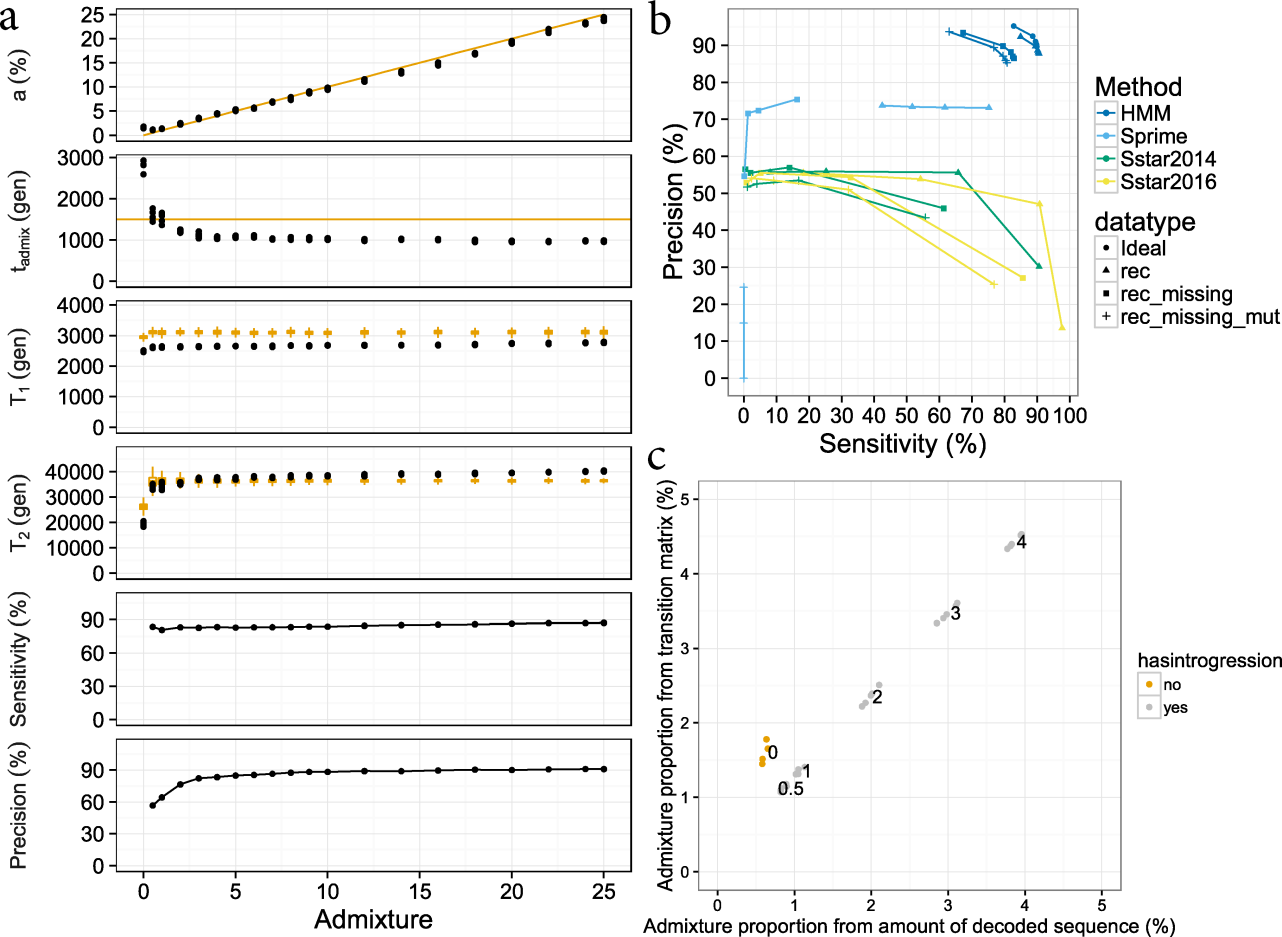
Evaluation of the model on simulated data. a). The estimated parameters *T*_*admix*_, *a*,*T*_*Ingroup*_ and *T*_*Archaic*_ are shown for different admixture proportions in simulated data with varying recombination rate and missing data. We also show the sensitivity and precision for different admixture proportions. For sensitivity and precision we show the values with a posterior probability cutoff at 0.5 (average posterior probability of all bins being belonging to the archaic state for a segments) b). Sensitivity and precision shown for the Sstar methods, Sprime and the HMM on different datasets. For Sstar and Sprime methods the different points are when the score for a segment is 50,000, 100,000, 150,000 and 200,000 as in Browning et al 2018. For the HMM the cutoffs is 0.5, 0.6, 0.7, 0.8 and 0.9. c) When there is no admixture the model is not in agreement with itself. The estimated admixture proportion from the transition matrix does not match the amount of sequence classified as belonging to the archaic state.

We estimated the transition and emission parameters using the Baum-Welch algorithm and used them to get an estimate for the admixture time *T*_*admix*_, the admixture proportion *a* and the mean coalescent times with the outgroup *T*_*Ingroup*_ and *T*_*Archaic*_ for the ingroup and archaic segments respectively. We also show the sensitivity and precision of the model at different admixture proportions. We only show the estimated parameters and error rates for simulations with missing data and varying recombination rate, because the addition of a varying mutation rate has a very minor effect, see Fig 2B. A table containing all parameters from the model and the corresponding demographic parameters are listed in S3 Dataset.

We evaluate the performance of the model in terms of precision (amount of predicted archaic sequence that is archaic/amount of predicted archaic sequence) and sensitivity (amount of predicted archaic sequence that is archaic/amount of true archaic sequence).

When admixture proportions are low (less than 2%) the model does not fit the emission or transition parameters well and the precision is around 70%. With an admixture proportion greater than 2% the model fits the parameters well with sensitivity above 80% and precision greater than 80% at a posterior probability cutoff at 0.5 (mean posterior probability of being archaic for all windows in segment). Raising the posterior probability cutoff from 0.5 to 0.8 increases the precision to around 90% while the sensitivity is still above 75% for 5% admixture as can be seen in S3 Fig. The effect of changing the cutoff for different admixture proportions are shown in S3 Dataset.

Across all scenarios *T*_*Ingroup*_ is slightly underestimated for data sets, while *T*_*Archaic*_ is overestimated, see Fig 2A. The error in estimating *T*_*Ingroup*_ and *T*_*Archaic*_ is likely due to model misspecification: the model effectively represents the distribution of coalescence times with the mean coalescence time only, and assumes all sites are heterozygous for both states. The effect of treating all sites as heterozygous is seen when comparing the *T*_*Ingroup*_ for haploid (mean = 2,952 generations ago) and diploid data (mean = 2,646 generations ago). The average simulated *T*_*Ingroup*_ was 3,109 generations ago. The effect is also seen when comparing *T*_*Archaic*_ for haploid (mean = 36,844 generations ago) and diploid data (mean = 38,508 generations ago). The average simulated *T*_*Archaic*_ was 36,462 generations ago.

Furthermore, the model tends to classify deeply coalescing haplotypes from the common ancestor of ingroup and outgroup as admixed. This effect is greater in simulations with low admixture proportions (Fig 2A).

The misspecification of the coalescent times is more problematic in cases where the ancestral population of ingroup and outgroup is very large and/or contains strong population structure. This can be overcome to some extent by sequencing more individuals from the outgroup, but the improvement becomes limited if there have been bottlenecks in the outgroup. The effective population size of the archaic source population after its separation from the common ancestor of ingroup and outgroup does not matter, since we assume very few lineages contribute to the admixture in this population that affects the test sample. Finally, a large population size in the ancestral population will increase the variance in coalescent times in the archaic state, but we would expect this to have less consequence on the models ability to discriminate.

We find that when admixture proportion are >5% and the recombination rate across the genome is constant the model recovers the right admixture time (S3 Dataset). However with varying recombination rate we underestimate the admixture time, which might be due to the model’s failure to identify around 80% of the short segments (see S2 Fig). This would increase the average segment length and lead to a more recent admixture time.

The admixture proportion is fitted well for all simulations except where the admixture proportion is zero. In this case, the “archaic” state is assigned to a set of segments with longer coalescent times to lineages in the outgroup, but their posterior probability is lower than for real admixed segments. The inconsistency between the estimated admixture proportion and the amount of segments recovered by posterior decoding, could potentially be used to discriminate whether or not there is admixture (Fig 2C). For admixture proportions below 2% (0.5% for data with varying recombination rate) we observe that *T*_*admix*_ is estimated to be greater than *T*_*Ingroup*_ (meaning admixture happened before the split of ingroup and outgroup), which is not possible and also indicates breakdown of the model.

We also compared the performance of our method to Sstar 2014[17], Sstar 2016[9] and Sprime 2018[13] under scenarios of varying recombination rate, varying recombination rate and missing data and varying mutation rate, missing data and varying mutation rate. Our method shows improved tradeoff between sensitivity and precision, because we take missing data into account when training and decoding the model, (Fig 2B and S3 Fig).

### Application to Papuan genomes

Having validated the model, we applied it to 14 Papuan individuals from the Simons Genome Diversity Project [18], 40 Papuans from [19] and an additional 35 Papuans [9]. We also analyzed individuals from West Eurasia, East Asia and South East Asia from the Simons Genome Diversity Project[18].

We note that variants in these datasets have been found using different bioinformatics pipelines with different filters but that the counts of heterozygous and homozygous variants are similar, see S4 Dataset.

We estimate the background mutation rate in windows of 100 kb, using the variant density of all variants in African populations from the 1000 Genomes Project.

Our model will not be able to distinguish Neanderthal from Denisova segments in Papuans, because the Denisovans and Neanderthals share a common ancestor before they do with humans and therefore the mean coalescence time with humans will be the same [1]. This means that the Poisson parameters will be the same as they both depend on *T*_*Archaic*_. However, we are able to enrich for Denisova versus Neanderthal segments by using different outgroups in our filtering step, because Neanderthal ancestry is common to all non-African populations whereas Denisovan ancestry relatively more private to Melanesia [7,9].

For each individual we used two different sets of variants as outgroup.

First, we used only variants found in Sub-Saharan African populations as an outgroup (A total of 324 individuals, where 292 individuals are from 1000 genomes and 32 individuals are from Simons diversity). This should remove variation in the common ancestor of Sub-Saharan Africans and the Papuans, retaining archaic variants of Neanderthal and Denisova origin as both are present in Papuans, but mainly absent in Africa [7,9]. We also used this filter when analyzing Eurasian populations.

Second we remove variants found in all non Papuan populations (A total of 2751 individuals, where 2504 individuals are from 1000 genomes and 247 individuals are from Simons diversity), only retaining variants that are unique to Papuan populations. This should remove Neanderthal variants that are shared with other non-African populations [1] and also to some extent remove variants of Denisovan origin that are found in Asians and Native Americans [20,21]. Thus removing all variants from the 1000 Genomes Project should enrich for Denisovan segments, while the segments that are found when using Sub-Saharan Africans but not using all 1000 Genomes Project samples as outgroups should be enriched for Neanderthal segments.

We estimated the optimal set of transition and emission parameters for each Papuan individual and found them to be largely consistent across the different datasets, see S4 Fig.

**Fig 3:**
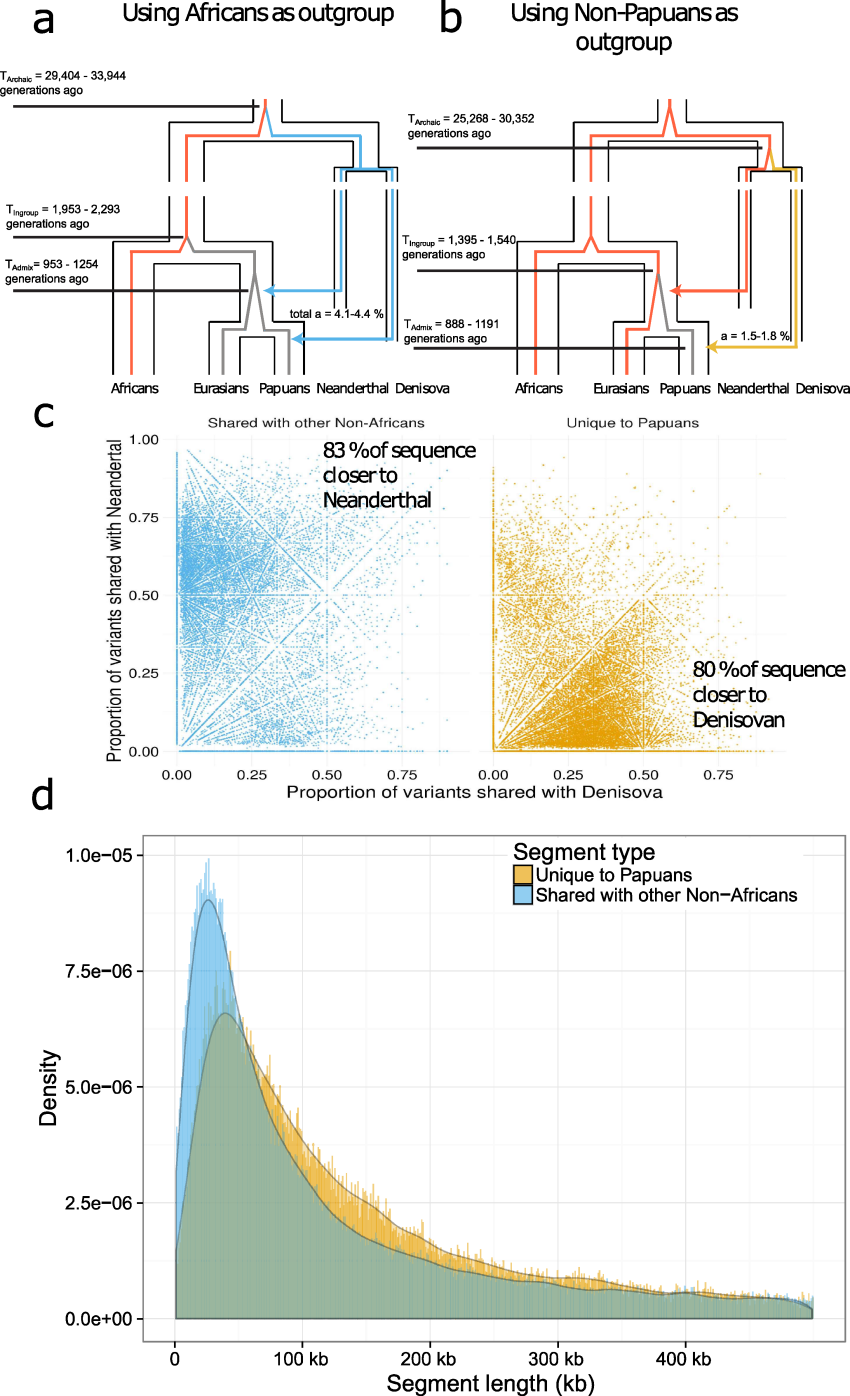
Application of model to Papuan genomes. a) Relationship between modern and archaic humans with the outgroup branches (Sub-Saharan Africans) colored in red. The average coalescence times for ingroup and outgroup *T*_*Ingroup*_ and archaic and outgroup *T*_*Archaic*_ are shown. The admixture proportions *a* and admixture time *T*_*admix*_ are shown for segments that are shared with other non-African populations. b) The outgroup colored in red is now all non-Papuans, and the new demographic parameters are shown. c) The segments that are shared with other Non-Africans share more variation with the Vindija Neanderthal than they do with the Altai Denisova. Segments that are unique to Papuan individuals share more variation with Altai Denisova than they do with the Vindija Neanderthal. d) Archaic segments that are shared with other non-African populations are shorter than segments that are unique to Papuans (segments with a mean posterior probability > 0.5 are kept).

The parameters were converted into estimates of *T*_*admix*_, *a*, *T*_*Ingroup*_ and *T*_*Archaic*_ using an average recombination rate of 1.2 ∙ 10^−8^ events per base pair per generation and an average mutation rate of 1.25 ∙ 10^−8^ mutations per base pair per generation, see Fig 3A and 3B. A table containing all parameters from the model and the corresponding demographic parameters is included in S4 Dataset.

We find that the mean coalescence time between Papuans and non-Papuan individuals is more recent (1,395–1,540 generations ago) than that between Papuans and Sub-Saharan Africans (1,953–2,293 generations ago) reflecting that Papuans are more closely related to other Non-Africans than to Africans. The mean coalescence time between Papuans and other non-Africans also provides an upper limit for Neanderthal introgression because it happened in their ancestral populations.

Using only Sub-Saharan individuals as an outgroup we find the mean coalescence time between the archaic and outgroup to be between 29,404 and 33,944 generations ago. When using non-Papuans as an outgroup the estimate is between 25,268 and 30,352 generations ago. The lower estimate is likely due to some variants in the common ancestor of Denisovans and Neanderthals having been removed in the latter case.

Using Sub-Saharan Africans as an outgroup we estimate the total admixture proportion of archaic sequence into Papuans to between 4.1–4.4% and the admixture proportion private to Papuans to be 1.5–1.8%. This means that approximately 2.6% is shared with non-Papuans, (Fig 3A).

From the transition parameters, we estimate that the admixture event with non-Africans happened 953–1,254 generations while the Papuan specific admixture event happened 888–1,191 generation ago. Both are likely underestimates as it was for the simulated data with missing data and varying recombination rate. Neanderthal admixture likely occurred closer to 2,000 generations ago after the out of Africa migration [7,22] with Denisovan admixture occurring after that.

We used a threshold of 0.8 posterior probability as it showed a good trade-off between precision and sensitivity on simulated data see Fig 2C and S3 Fig. By comparing to the Vindija Neanderthal [10] and Denisova [2] genomes we find that this cutoff removes around 65% of the segments that don’t share variants with any archaic reference genome that were found with a cutoff of 0.5, while only removing 10.4% of the total length of archaic segments, see S5 Fig. Short segments that do not share variants with any archaic reference genome may be enriched for false positive variant calls, or may be deeply coalescing modern human haplotypes, in addition to the possibility of containing material from an unknown archaic source. We note that all segments with very high confidence share variants with Neanderthal and/or Denisova references.

When we use a cutoff of 0.8 we find that 84% of the segments unique to Papuans (80% of the total sequence) shared more variants with the Denisova genome than with the Vindija Neanderthal, and that 78% the segments that are shared with other non-Africans (83% of the total sequence) shared more variants with the Vindija Neanderthal than the Denisova (Fig 3C). This is consistent with most archaic sequence unique to Papuans coming from Denisovans, and most shared archaic sequence coming from Neanderthals.

However, segments that are unique to Papuans are longer on average (94.2 kb) compared to those shared with other non-African populations (76.9 kb), see Fig 3D. The differences in length distributions are not seen as clearly when using Sstar, Sprime or CRF, see S6 Fig. Moreover, the length distributions of archaic segments that are not unique to Papuans (putative Neanderthal segments) are more similar to those found in other non-African populations, see S7 Fig, consistent with a single Neanderthal admixture event.

We compared our archaic segments to those previously reported using other methods [7,9,13]. We find that our method recovers 67% of the archaic sequence found using CRF, 84.9% of the archaic sequence found using Sprime and 74% of the archaic sequence found using Sstar.

When comparing the detected segments to the archaic reference genomes our method finds more Denisova segments in Papuans than Neanderthal ones, unlike Sstar and CRF. Our method also detects a smaller amount of additional Denisova segments in East and South East Asians, (Table 1). A dataset with all inferred segments from Papuans and Simons Genome Diversity Project individuals can be found in S5 Dataset.

**Table 1:** Amount of sequence of different origins. For different methods and populations, the amounts of sequence (in Mb) are shown in putative archaic segments that share equal numbers of private variants with the Denisova and Vindija Neanderthal (Both), more with Denisova, none with either, or more with Vindija Neanderthal. Neither Sstar nor CRF label segments that do not share variants with the archaic reference genomes. For CRF, segments had to be either more similar to Neanderthal than Denisova or vice versa so they do not report segments that match both equally well. For Sstar the comparison to Denisova was only made for Papuans. Note the Papuans individuals used in Sstar are admixted with East Asians.

*Method*	*Population*	*Both*	*Denisova*	*None*	*Neanderthal*	*Total*
HMM	Papuan	4.35	83.11	11.54	71.70	170.7
	eastasia	1.48	5.69	9.96	61.37	78.49
	southasia	1.62	5.85	10.12	51.36	68.95
	westeurasia	1.47	2.39	10.14	43.95	57.94
Sstar	Papuan	26.5	43.11	-	49.21	118.82
	eastasia	-	-	-	65.02	65.02
	southasia	-	-	-	55.18	55.18
	westeurasia	-	-	-	51.23	51.23
CRF	Papuan	-	58.17	-	84.72	142.89
	eastasia	-	3.21	-	72.92	76.14
	southasia	-	2.79	-	61.36	64.15
	westeurasia	-	0.68	-	57.29	57.97
Sprime	Papuan	1.04	38.98	13.48	27.85	81.36
	eastasia	0.89	4.29	14.14	60.49	79.81
	southasia	0.76	4.60	15.09	53.83	74.29
	westeurasia	0.76	1.68	14.02	52.22	68.70

## Discussion

Our method examines the number of private variants in the ingroup compared to the outgroup and implicitly, the distance between the ingroup and the outgroup haplotypes, which interestingly are the features found to carry the highest weights in the new method of Durvasula et al.[14]. Our implementation also allows for missing data, unlike Sprime and Sstar, which may potentially be useful for analysis of ancient DNA samples.

Since emission probabilities are very different between the human and archaic states in our model, we expect a low rate of false positive archaic inference, and this is also what we see in simulations. However, since recombination rates are highly variable, we expect many very short archaic segments and these have a high false negative rate. Our inability to identify these causes us to underestimate the admixture time. Nevertheless, the model does recover the correct size distribution for longer segments (> 50 kb), (S2 Fig). The mean coalescence times of modern and archaic humans are reasonably well estimated in simulations. One issue of interest is that if there were additional super-archaic introgression into the sequenced Denisovan as previously proposed [1], this would cause the mean coalescence time in Denisovan introgressed segments to be greater than that for Neanderthal segments. We did not observe this, although we note that there may be confounding from a low level of Denisovan admixture also present in East Asians which form part of our contrast population, reducing the observed mean divergence.

We report more Denisova segments than approaches relying on the Denisovan reference. This is possibly because our method does not rely on matching to the Altai Denisova sequence, which is believed to be considerably diverged from the source population for “Denisovan” admixture into Papuan ancestors, probably shortly after the split of Denisovans from Neanderthals [1]. Furthermore, because of this early split, many segments may be equally close to the Vindija Neanderthal and the sequenced Denisova sample, and we expect that a fraction of segments introgressed from the Denisovan are more closely related to Vindija and vice versa due to incomplete lineage sorting.

We find no clear evidence for an introgression with a new archaic hominin in Papuans, but we do find segments that do not share variation with any of the sequenced archaic populations. These segments could represent variation in Neanderthals and Denisovans that is not captured by the three high coverage archaic reference genomes, or another source. In the future it will be interesting to compare these segments to other human populations that might also have archaic segments of unknown origin [11,12].

Our model is not restricted to being applied to humans. We have called the admixture source “archaic” so far, which is standard in the human context, but more generally we are modelling a particular form of population structure involving an admixture event from a distantly diverged lineage. We note that other types of population structure, for example involving continual gene flow, could also create signals under our model. Subject to this caveat, the method can be applied where samples have been sequenced from a population that is hypothesized to have received admixture from a perhaps unknown source, and there is comparable data from an outgroup population that did not receive the admixture. The performance of the method depends on the ratio of signal to noise. The signal is stronger the more admixture there is, the more divergent the admixture source is, and the more recently the admixture happened. The noise increases if the outgroup diverged longer ago from the test samples, and if the common ancestor of the ingroup and outgroup had larger population size. The latter problem can be mitigated by sequencing more individuals from the outgroup. Therefore, as an increasing number of individuals are being sequenced in other species, our method could be used to explore introgression in those species, for example chimp and bonobo [23], bears [24], elephants [25] or gibbons [26].

## Materials and methods

### Simulations

To simulate data we used Msprime [27]. We simulated 5 Papuans and as an outgroup we simulated 500 Africans, 100 Europeans and 100 Asians using demographic parameters from [19]. We simulated data where we varied the recombination rate according to HapMap recombination maps [16] for 5 individuals and removed variants within non-callable regions and variants that were found in the simulated outgroup. We grouped all autosomes into bins of 1000 base pairs and counted the number of variants. For each 1000 bp window we calculated the number of called bases using the repeat masked segments.

We simulated 22 autosomes with varying mutation rate in segments with a mean length of 1 Mb across the genome. The mutation rate in a segment could either be 1.25*10^-8 mutations per base-pair per generation–the average rate, 50% decrease of the average rate or 50% increase of the average rate. We picked the mutation rate in each segment randomly. The choice of segment length and mutation rate is based on [28].

### Train parameters and decode segments

We trained and decoded the segments using our HMM, which is available at: https://github.com/LauritsSkov/Introgression-detection/

### Data sets

We used 14 Papuans, 71 WestEurasians, 72 East Asians and 39 South Asians individuals from the Simons Genome Diversity Project (SGDP) [18], 40 Papuans from [19] and an additional 35 Papuans [9].

### Filtering variants in real data

We used two sets of outgroups. One is all Sub-Saharan Africans (populations: YRI, MSL, ESN) from the 1000 Genomes Project [29] and all Sub-Saharan African populations from SGDP [18] except Masai, Somali, Sharawi and Mozabite, which show signs of out-of-Africa admixture. The other outgroup is all individuals from the 1000 Genomes Project [29] plus all non-Papuans from SGDP. For all human data sets, we also removed sites that fell within repeatmasked [15] regions, and sites that were not in the strict callability mask for the 1000 Genomes Project.

### Repeat mask regions

hgdownload.cse.ucsc.edu/goldenpath/hg19/bigZips/chromFaMasked.tar.gz

Strict callability mask for 1000 genomes: ftp://ftp.1000genomes.ebi.ac.uk/vol1/ftp/release/20130502/supporting/accessible_genome_masks/StrictMask/

The background mutation rate was calculated using the density of all variants from populations YRI, LWK, GWD, MSL and ESN in windows of 100 Kb divided by the mean variant density of the whole genome.

### Comparison to Sstar, Sprime and Conditional Random Field

We called Neanderthal and Denisova segments in the 14 Papuans and compared them to the segments called with CRF with more than 50 posterior probability [7] available at: **https://sriramlab.cass.idre.ucla.edu/public/sankararaman.curbio.2016/**.

The path to the haplotypes is: summaries/2/denisova/oceania/summaries/haplotypes/CRHOM.thresh-50.length-0.00.haplotypes.

We called Neanderthal and Denisova segments in the 35 Papuans and compared them to the segments called with Sstar with more than 99 posterior probability [9] available at: https://drive.google.com/drive/folders/0B9Pc7_zItMCVWUp6bWtXc2xJVkk

The path to the haplotypes is: introgressed_haplotypes/LL.callsetPNG.mr_0.99.den_calls_by_hap.bed.merged.by_chr.bed

We called Neanderthal and Denisova segments in the 14 Papuans and compared them to the segments called with Sprime with a score greater than 150,000 [13]. The path to the data is: https://data.mendeley.com/datasets/y7hyt83vxr/1

## Supporting information

S1 Fig Demographic parameters for simulation. The effective population sizes, split times and bottleneck population sizes are shown for the simulated populations.
(PDF)

S2 Fig Total segments and sequence called SIM. The first column show the total number of segments found and the second column show the total amount of sequence that these segments add up to. The rows are different simulation scenarios and the colors of the stacked bar plot show the amount/number of segments that are not found using posterior decoding, where less than half of the segment overlap with the true archaic segments or where more than half of the segment overlaps with the true archaic segment.
(PDF)

S3 Fig Effect of adjusting cutoff for when to include a putative archaic segment. The amount of sensitivity and precision found when the posterior cutoff is varied. The different colors are different simulation scenarios; ideal is simulated data with constant recombination rate and no missing data, ideal_hap is the same dataset but haploid genomes are used, rec is simulated data where the recombination rate varying along the genome, rec_missing is simulated data with varying recombination rate and missing data and rec_missing_mut is simulated data with varying recombination rate, missing data and varying mutation rate. The admixture proportion for all data is 5%.
(PDF)

S4 Fig Parameter estimation of Papuans. The different subpanels show the estimates for the parameters t_admix, a, T_ingroup and T_archaic depending on which outgroup was used (Sub-Saharan Africans) or the whole world (non-Papuans). There is a separate bar for each individual, and the bars are colored according to which dataset they came from.
(PDF)

S5 Fig Segment distributions as a function of posterior probability. Distributions of the number (left) and total length (right) of segments with mean posterior probability as on the x axis. Numbers are given for all 89 Papuans, called with Sub-Saharan Africans as the outgroup, and with a threshold of 0.5.
(PDF)

S6 Fig Length distribution of inferred segments for other methods. The length distribution of all Denisova and Neanderthal segments found using conditional random field (CRF), the hidden Markov model (HMM) and Sstar. For our HMM, Neanderthal are those segments that are shared with other non-African populations and Denisova are those unique to Papuans.
(PDF)

S7 Fig Length distribution of Asians, Europeans and Papuans. The length distributions of segments unique to Papuans (Denisova) and segments shared with other non-African populations (Neanderthal) are shown for segments found using four different population groups.
(PDF)

S1 Dataset Converting model parameters to demographic parameters for haploid and diploid data. (PDF)

S2 Dataset Python script to simulate data. The python script is using the Msprime package to simulate whole genome sequences under a given scenario.
(PY) Click here for additional data file.

S3 Dataset This spreadsheet contains two tabs. **In the tab “Simulated data parameters” a table with each of the 15 simulated individuals.** For each individual the parameter estimates from the model and the corresponding demographic parameter is shown. In the tab “Simulated data precision_sens” the precision and sensitivity are shown for each individual with different cutoffs.
(XLSX)

S4 Dataset This spreadsheet contains a table with each of the 89 Papuans and the 182 individuals from Simons diversity project. For each individual the parameter estimates from the model and the corresponding demographic parameter is shown.
(XLSX)

S5 Dataset This table contains the inferred archaic segments for the 89 Papuans and the 182 individuals from Simons diversity project. The columns are shown in the header.
(TXT)
