# Supplementary material for: Detecting archaic introgression using an unadmixed outgroup
Source: PLoS Genet. 2018 Sep 18;14(9):e1007641. doi: 10.1371/journal.pgen.1007641 (PMC6161914; doi:10.1371/journal.pgen.1007641)
Supplement: S1 Fig — The effective population sizes, split times and bottleneck population sizes are shown for the simulated populations. (PDF) [file pgen.1007641.s001.pdf]

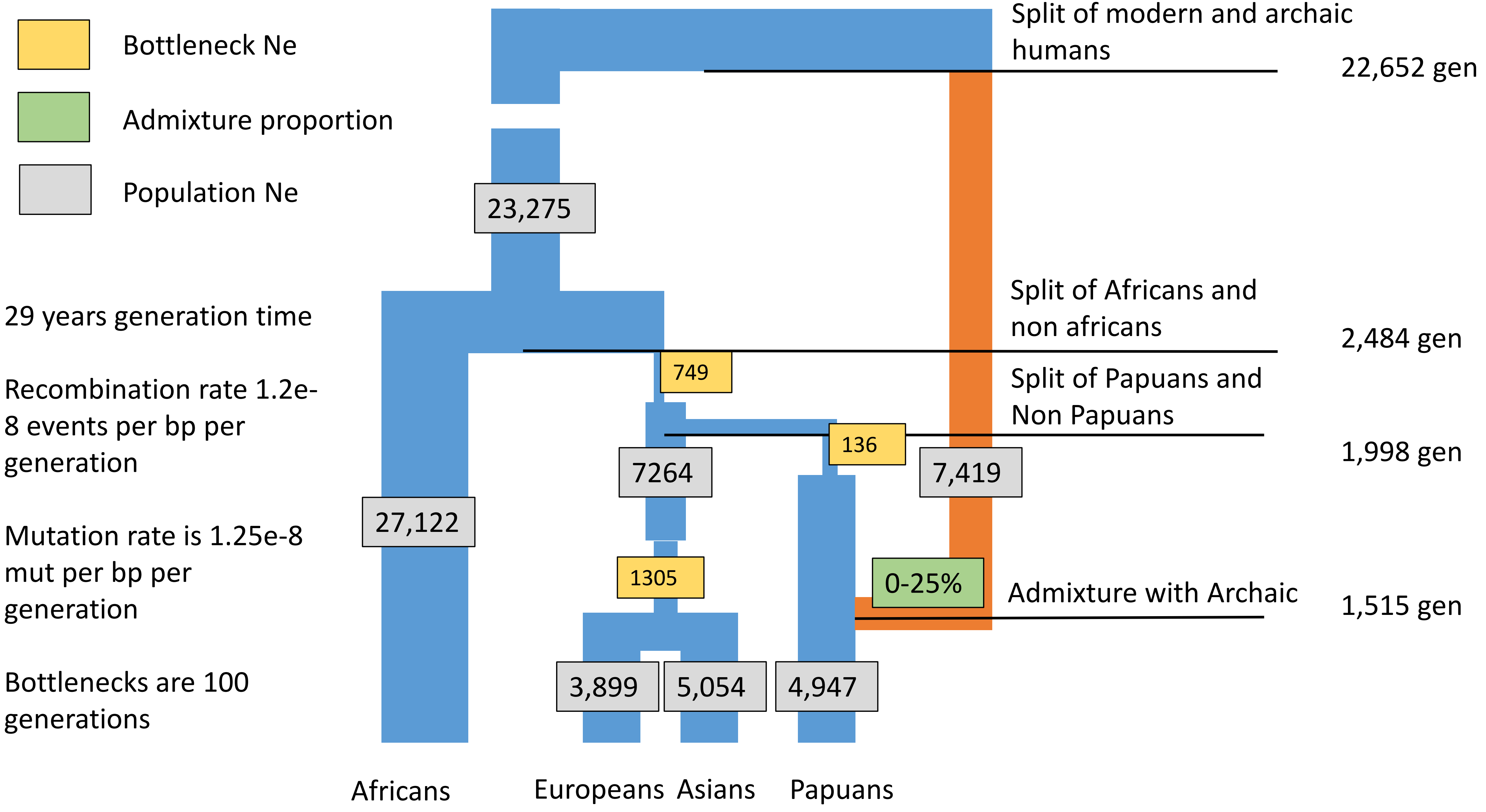
Bottleneck Ne
Admixture proportion
Population Ne
29 years generation time
Recombination rate 1.2e-8 events per bp per generation
Mutation rate is 1.25e-8 mut per bp per generation
Bottlenecks are 100 generations
Split of modern and archaic humans
22,652 gen
23,275
Split of Africans and non africans
2,484 gen
749
Split of Papuans and Non Papuans
1,998 gen
136
7264
7,419
27,122
1305
0-25%
Admixture with Archaic
1,515 gen
3,899
5,054
4,947
Africans
Europeans
Asians
Papuans
