# Supplementary figures and images for: Detecting archaic introgression using an unadmixed outgroup
Source: PLoS Genet. 2018 Sep 18;14(9):e1007641. doi: 10.1371/journal.pgen.1007641 (PMC6161914; doi:10.1371/journal.pgen.1007641)

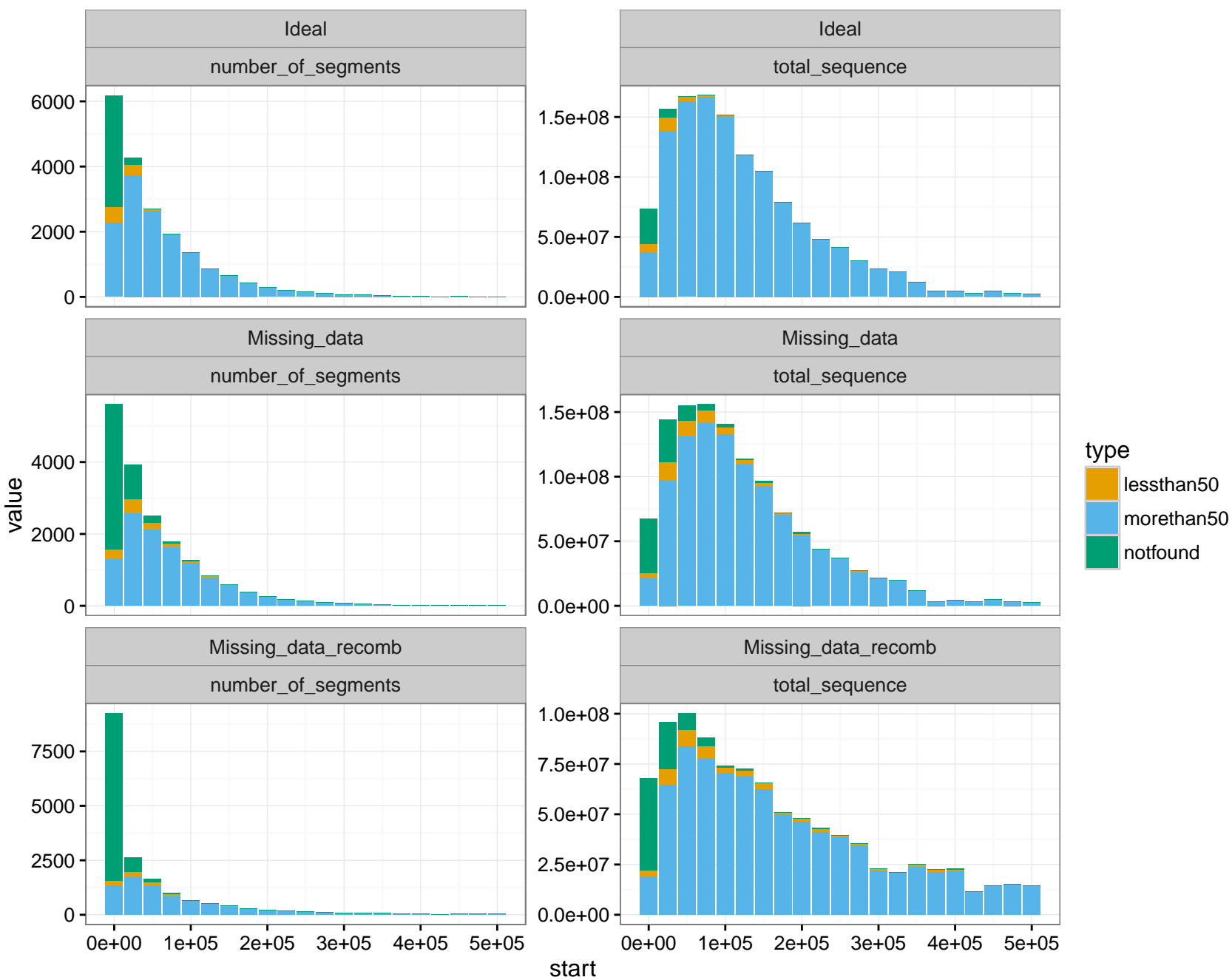

Supplement: S2 Fig — The first column show the total number of segments found and the second column show the total amount of sequence that these segments add up to. The rows are different simulation scenarios and the colors of the stacked bar plot show the amount/number of segments that are not found using posterior decoding, where less than half of the segment overlap with the true archaic segments or where more than half of the segment overlaps with the true archaic segment. (PDF) [file pgen.1007641.s002.pdf]

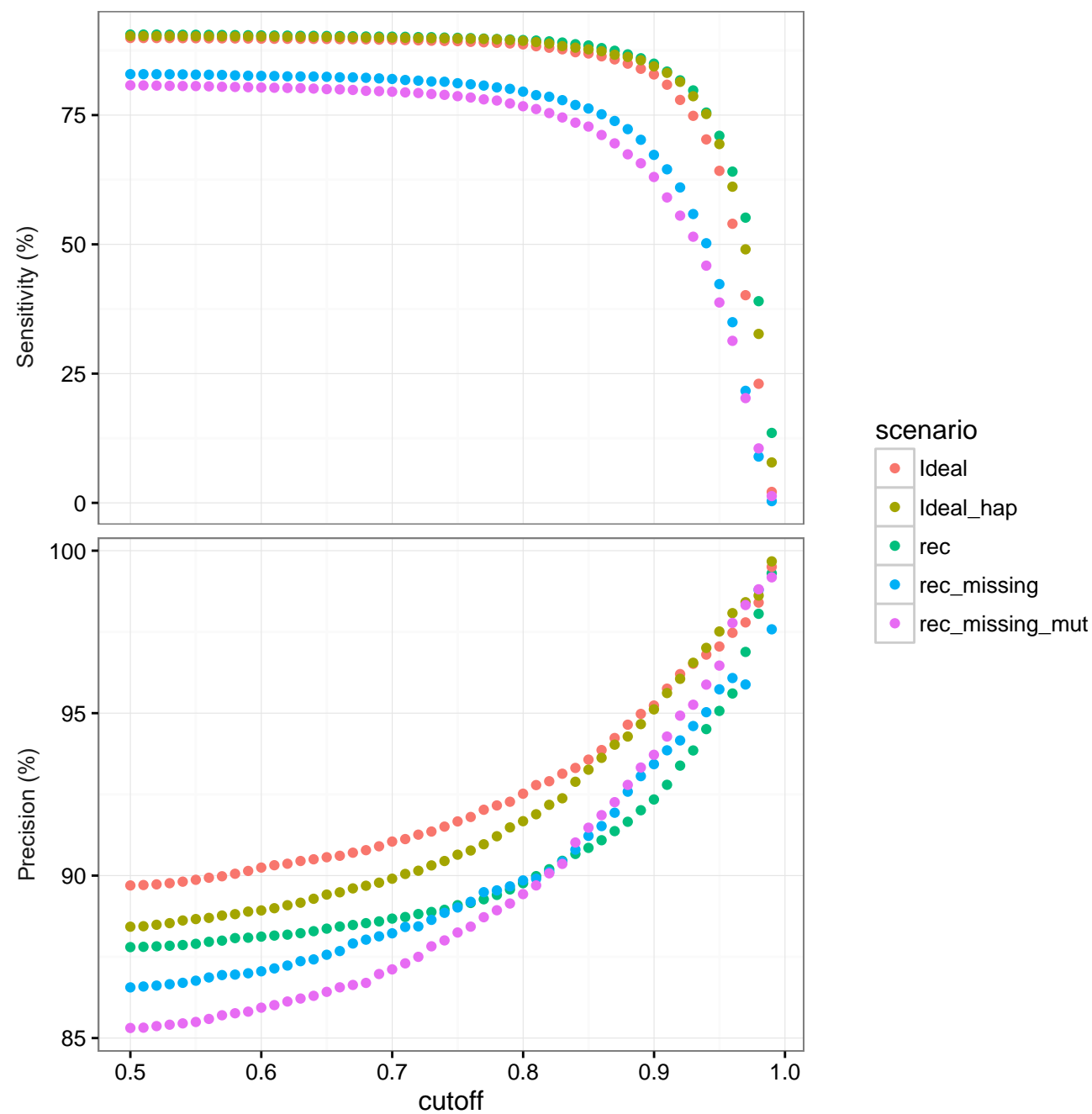

Supplement: S3 Fig — The amount of sensitivity and precision found when the posterior cutoff is varied. The different colors are different simulation scenarios; ideal is simulated data with constant recombination rate and no missing data, ideal_hap is the same dataset but haploid genomes are used, rec is simulated data where the recombination rate varying along the genome, rec_missing is simulated data with varying recombination rate and missing data and rec_missing_mut is simulated data with varying recombination rate, missing data and varying mutation rate. The admixture proportion for all data is 5%. (PDF) [file pgen.1007641.s003.pdf]

Subsaharan Africans

Whole world

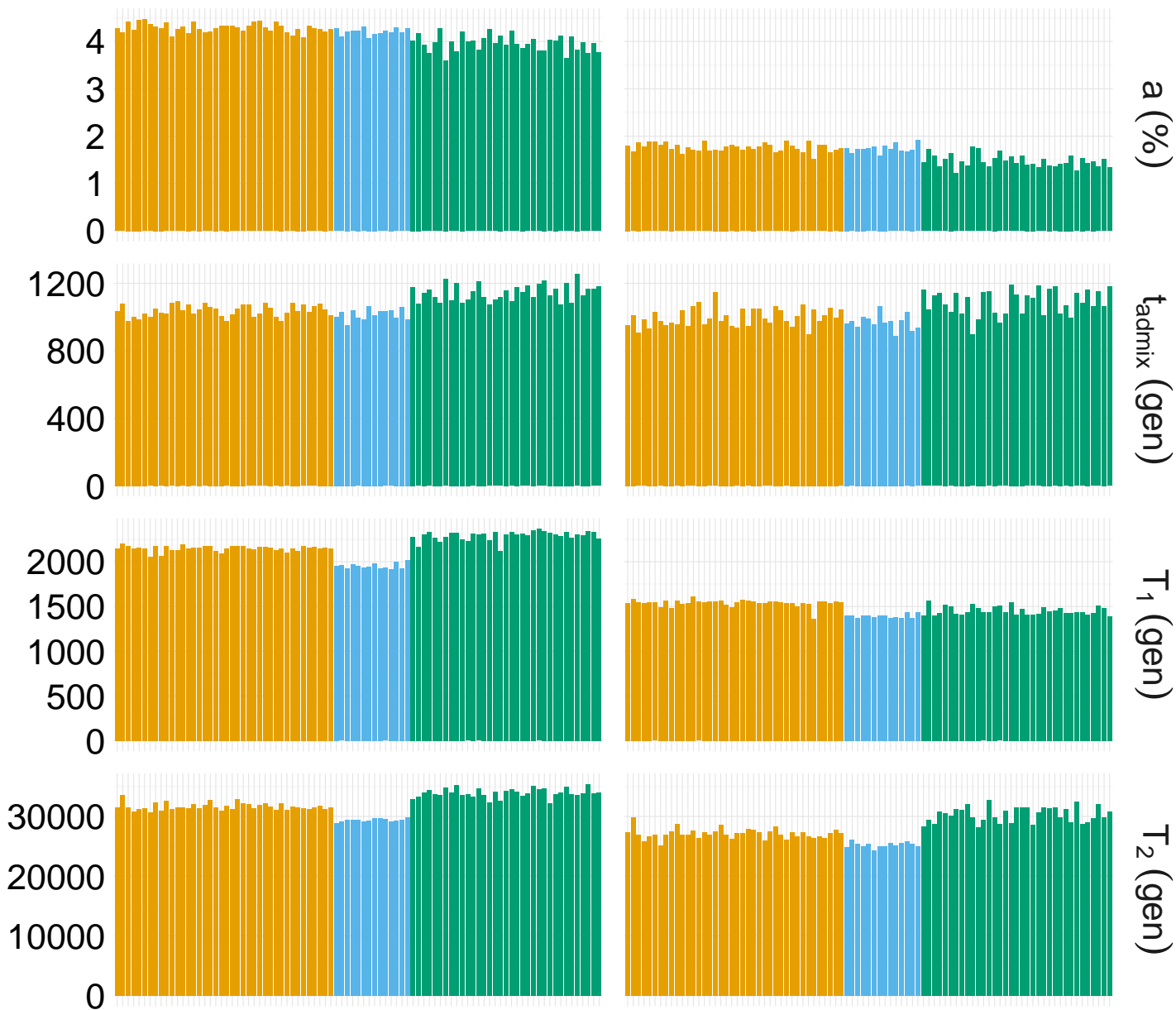

Dataset

Malaspinas2016

Sriram2016

Vernot2016

Supplement: S4 Fig — The different subpanels show the estimates for the parameters t_admix, a, T_ingroup and T_archaic depending on which outgroup was used (Sub-Saharan Africans) or the whole world (non-Papuans). There is a separate bar for each individual, and the bars are colored according to which dataset they came from. (PDF) [file pgen.1007641.s004.pdf]

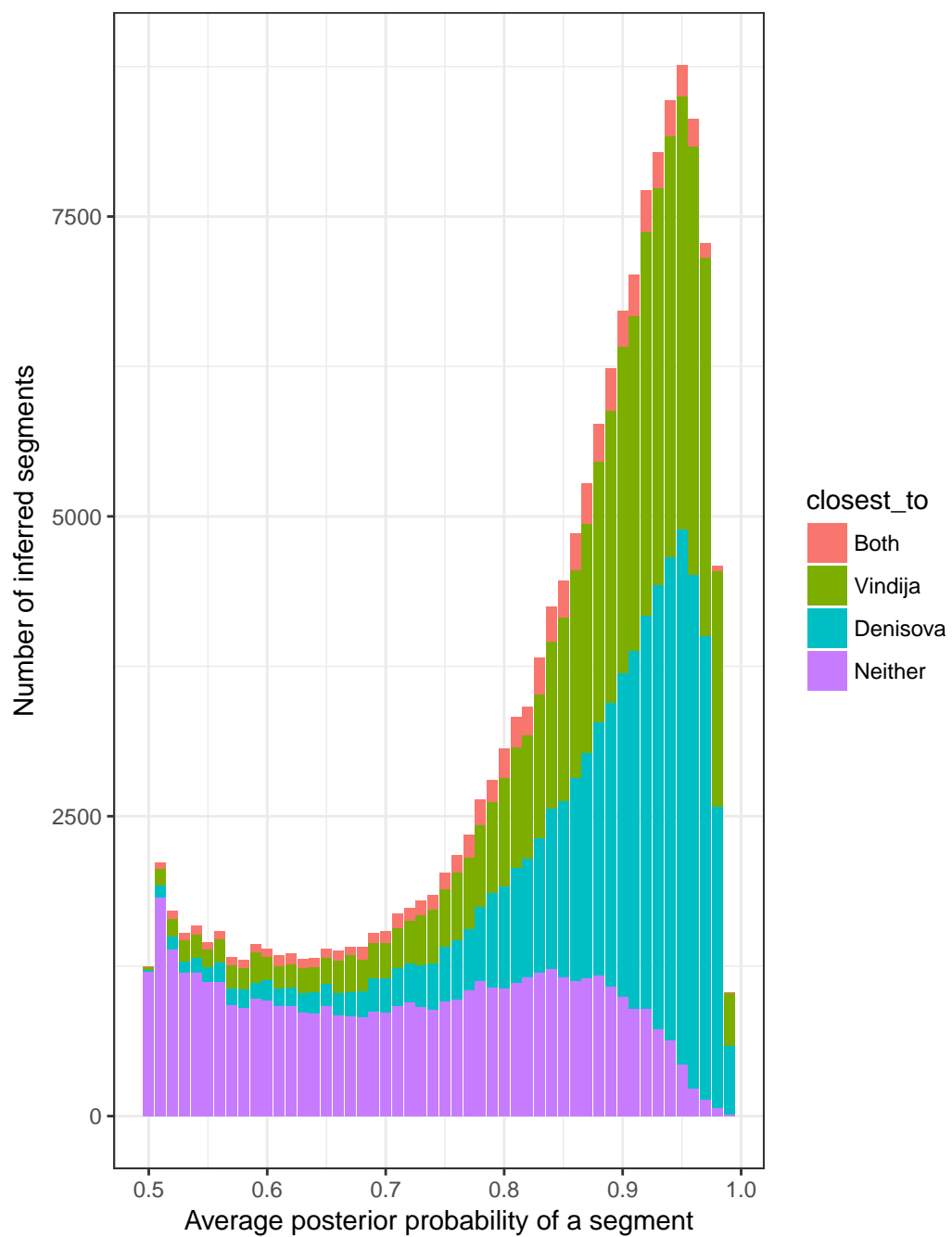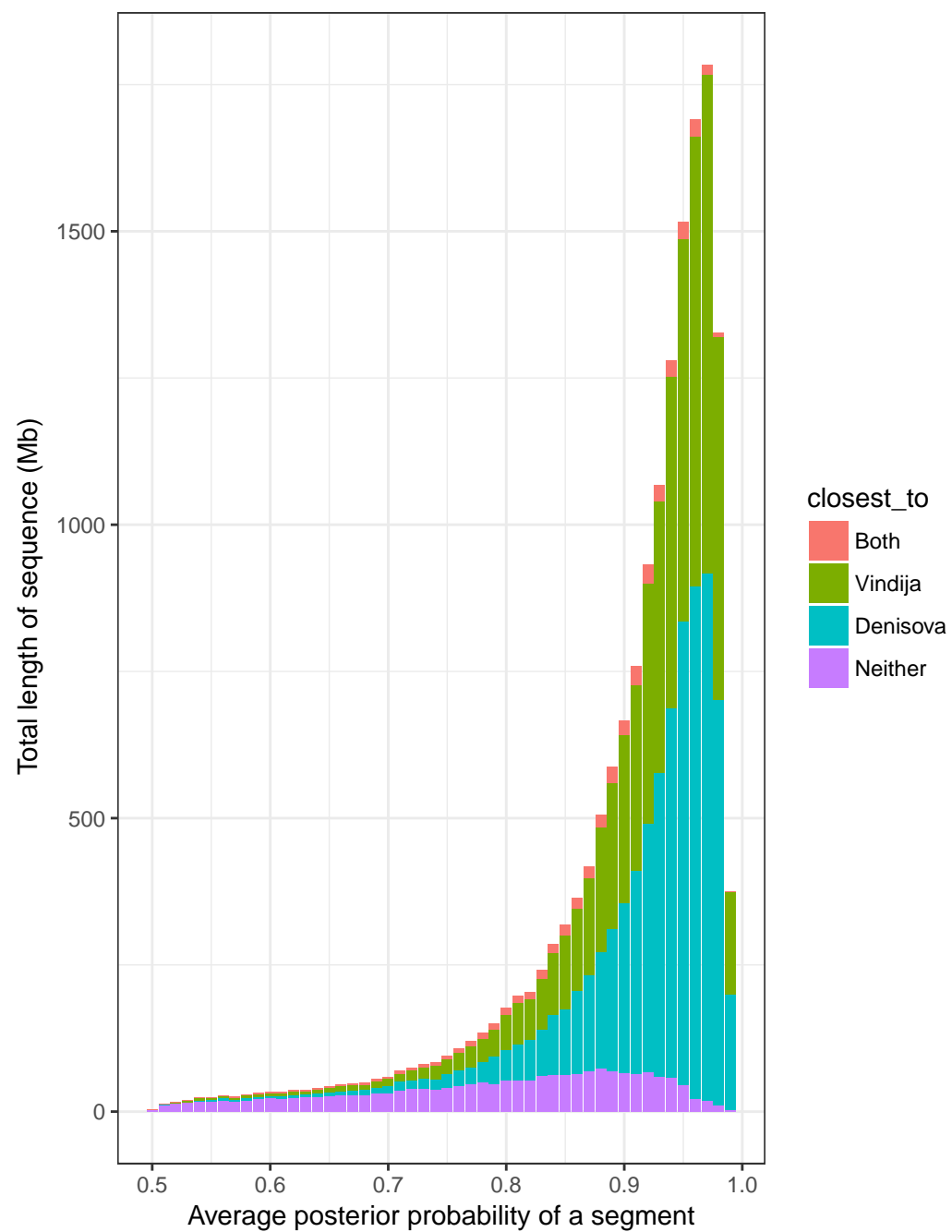

Supplement: S5 Fig — Distributions of the number (left) and total length (right) of segments with mean posterior probability as on the x axis. Numbers are given for all 89 Papuans, called with Sub-Saharan Africans as the outgroup, and with a threshold of 0.5. (PDF) [file pgen.1007641.s005.pdf]

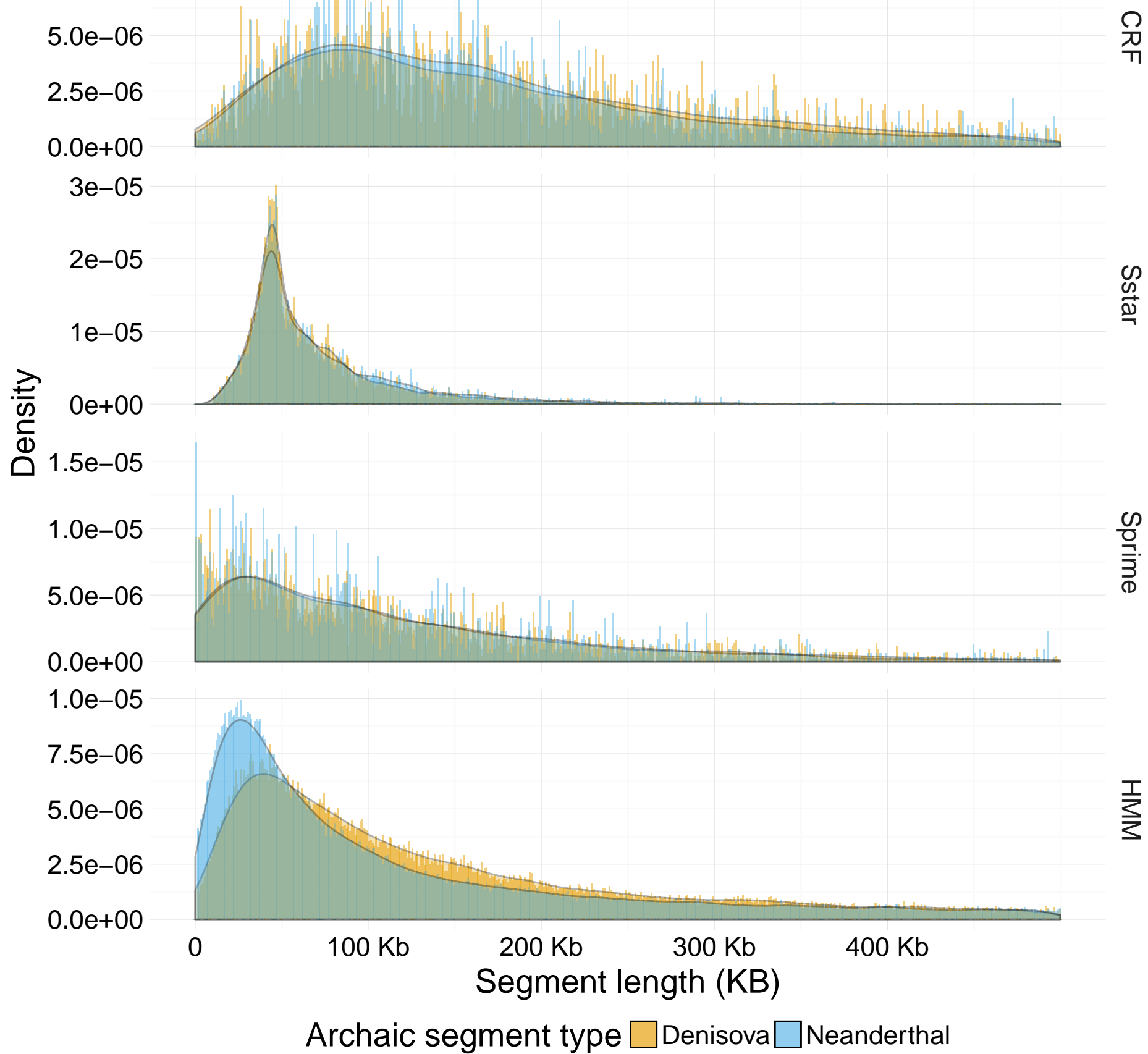

Supplement: S6 Fig — The length distribution of all Denisova and Neanderthal segments found using conditional random field (CRF), the hidden Markov model (HMM) and Sstar. For our HMM, Neanderthal are those segments that are shared with other non-African populations and Denisova are those unique to Papuans. (PDF) [file pgen.1007641.s006.pdf]
