# Supplementary material for: Detecting archaic introgression using an unadmixed outgroup
Source: PLoS Genet. 2018 Sep 18;14(9):e1007641. doi: 10.1371/journal.pgen.1007641 (PMC6161914; doi:10.1371/journal.pgen.1007641)
Supplement: S7 Fig — The length distributions of segments unique to Papuans (Denisova) and segments shared with other non-African populations (Neanderthal) are shown for segments found using four different population groups. (PDF) [file pgen.1007641.s007.pdf]

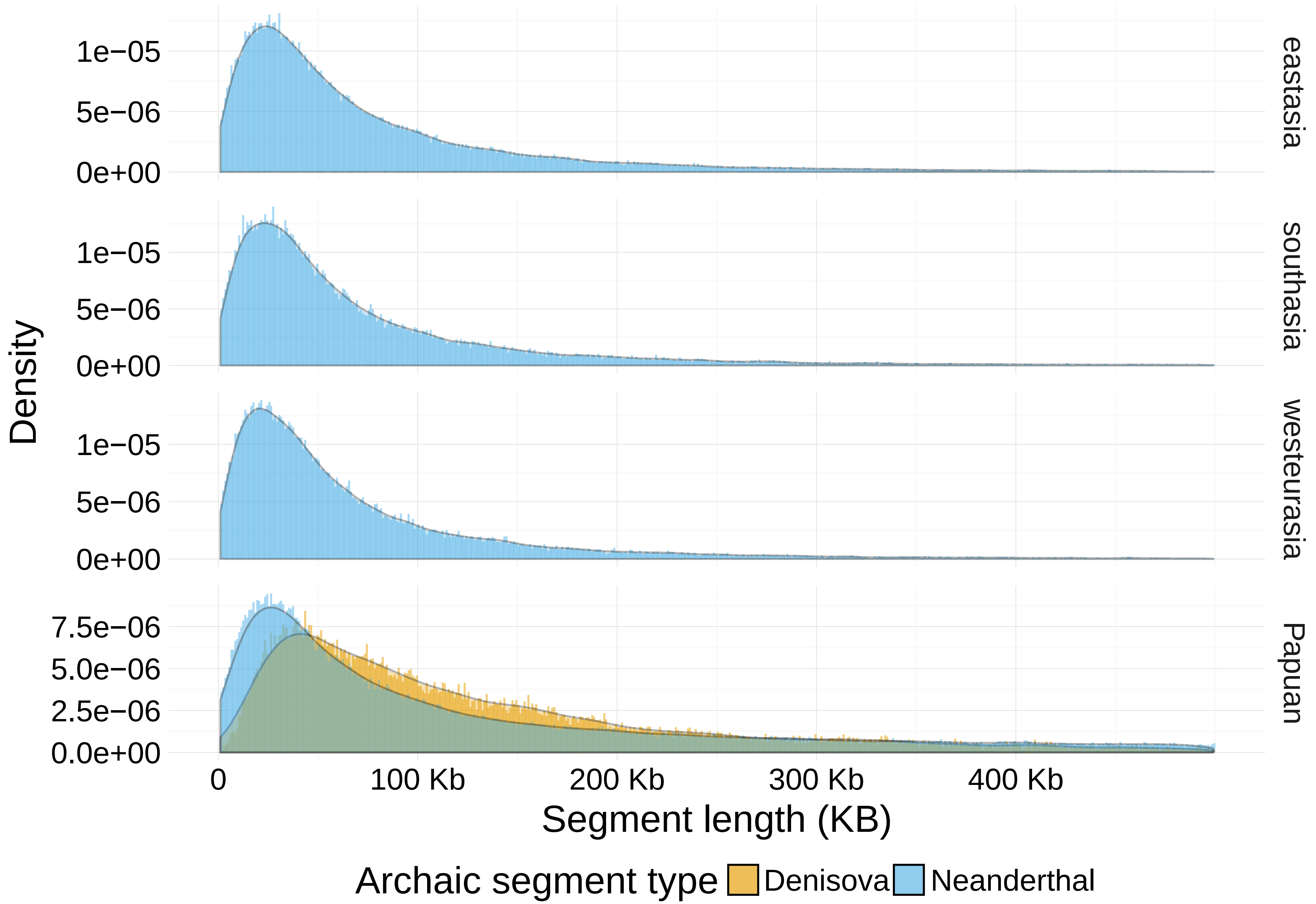
Density
1e−05
5e−06
0e+00
eastasia
southasia
westeurasia
7.5e−06
5.0e−06
2.5e−06
0.0e+00
Papuan
0
100 Kb
200 Kb
300 Kb
400 Kb
Segment length (KB)
Archaic segment type
Denisova
Neanderthal
