# Supplementary material for: Detecting archaic introgression using an unadmixed outgroup
Source: PLoS Genet. 2018 Sep 18;14(9):e1007641. doi: 10.1371/journal.pgen.1007641 (PMC6161914; doi:10.1371/journal.pgen.1007641)
Supplement: S1 Dataset — (PDF) [file pgen.1007641.s008.pdf]

Transition matrix

Ingroup to Ingroup and Ingroup to Archaic

Haploid case

Ingroup	Ingroup

$$P(I \rightarrow I) = e^{-T_{admix} \cdot r \cdot L} + (1 - e^{-T_{admix} \cdot r \cdot L})(1 - a)$$

No recombination

Recombination with another Ingroup segment

When $T_{admix} \cdot r \cdot L$ is small we can approximate

$$e^{-T_{admix} \cdot r \cdot L} \approx 1 - T_{admix} \cdot r \cdot L$$

$$\boxed{P(I \rightarrow I) = 1 - a \cdot T_{admix} \cdot r \cdot L}$$

The probability of changing state to the archaic state is then:

$$\boxed{P(I \rightarrow A) = a \cdot T_{admix} \cdot r \cdot L}$$

Diploid case

Ingroup	Ingroup
Ingroup	Ingroup

$$P(I \rightarrow I) = e^{-T_{admix} \cdot r \cdot L} + (1 - e^{-T_{admix} \cdot r \cdot L})(1 - 2a)$$

No recombinaiton

Recombination with another human segment

The 2a is because we dont want to recombine with an archaic segment on either chromosome. The rest of the calculations are the same.

$$\boxed{P(I \rightarrow I) = 1 - 2a \cdot T_{admix} \cdot r \cdot L}$$

$$\boxed{P(I \rightarrow A) = 2a \cdot T_{admix} \cdot r \cdot L}$$

Transition matrix

Archaic to archaic and archaic to Ingroup

Haploid case

Archaic	Archaic

$$P(A \rightarrow A) = e^{-T_{admix} \cdot r \cdot L} + (1 - e^{-T_{admix} \cdot r \cdot L})a$$

No recombinaiton

Recombination with another archaic segment

$$P(A \rightarrow A) = 1 - (1 - a) \cdot T_{admix} \cdot r \cdot L$$

$$P(A \rightarrow I) = (1 - a) \cdot T_{admix} \cdot r \cdot L$$

Diploid case

Archaic	Archaic
Ingroup	Ingroup

$$P(A \rightarrow A) = e^{-T_{admix} \cdot r \cdot L} + (1 - e^{-T_{admix} \cdot r \cdot L})a$$

No recombinaiton

Recombination with another human segment

If we assume that Archaic segments are only heterozygous the transition probabilities do not change.

$$P(A \rightarrow A) = 1 - (1 - a) \cdot T_{admix} \cdot r \cdot L$$

$$P(A \rightarrow I) = (1 - a) \cdot T_{admix} \cdot r \cdot L$$

Emission values

Ingroup and Archaic

Haploid case

Ingroup state

$$\lambda_{Ingroup} = \mu \cdot L \cdot T_{ingroup}$$

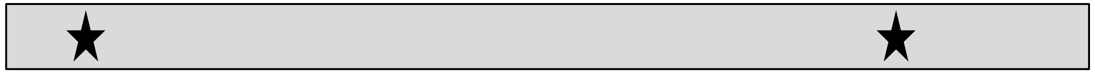

Archaic state

$$\lambda_{Archaic} = \mu \cdot L \cdot T_{Archaic}$$

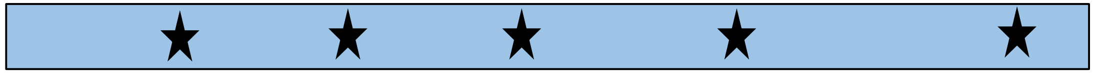

Diploid case

Ingroup state

$$\lambda_{Ingroup} = 2 \cdot \mu \cdot L \cdot T_{ingroup}$$

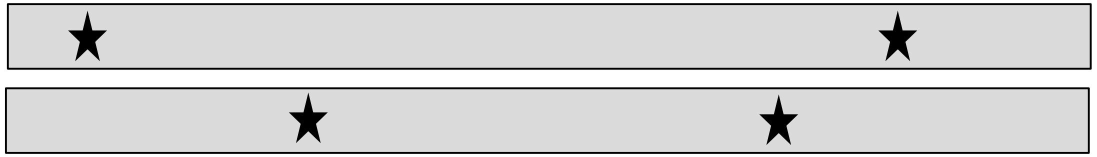

Archaic state

$$\lambda_{Archaic} = \mu \cdot L \cdot T_{Archaic} + \mu \cdot L \cdot T_{ingroup}$$

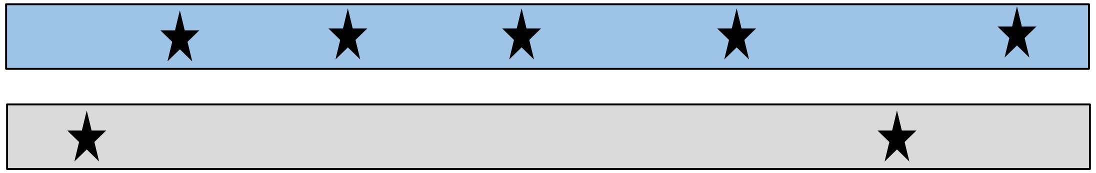

We assume archaic segments are always heterozygous
